# Trainee Surgeons Affect Operative Time but not Outcome in Minimally Invasive Total Hip Arthroplasty

**DOI:** 10.1038/s41598-017-06530-3

**Published:** 2017-07-21

**Authors:** Markus Weber, Achim Benditz, Michael Woerner, Daniela Weber, Joachim Grifka, Tobias Renkawitz

**Affiliations:** 1 Department of Orthopedic Surgery, Regensburg University, Medical Centre, Bad Abbach, Germany; 2 Department of Haematology and Oncology, Regensburg University, Medical Centre, Regensburg, Germany

## Abstract

Training of young surgeons in total hip arthroplasty (THA) is crucial, but might affect operative time and outcome especially in minimally invasive (MIS) THA. We asked whether the learning curve of orthopaedic residents trained on MIS THA has an impact on (1) operative time (2) complication rates and (3) early postoperative outcome. In a retrospective analysis of over 1000 MIS THAs from our institutional joint registry, operative time, complication rates, patient reported outcome measures (Western Ontario and McMaster Universities Arthritis Index [WOMAC] and Euro-Qol 5D-5L [EQ-5D]) within the first year and responder rates for positive outcome as defined by the Outcome Measures in Rheumatology and Osteoarthritis Research Society International consensus responder (OMERACT-OARSI) criteria were compared between trainee and senior surgeons. Mean operative time was nine minutes longer for trainees compared to senior surgeons (78.1 ± 25.4 min versus 69.3 ± 23.8 min, p < 0.001). Dislocation (p = 0.21), intraoperative fracture (p = 0.84) and infection rates (p = 0.58) were comparably low in both groups. Both trainee and senior THAs showed excellent improvement of EQ-5D (0.34 ± 0.26 versus 0.32 ± 0.23, p = 0.40) and WOMAC (45.9 ± 22.1 versus 44.9 ± 20.0, p = 0.51) within the first year after surgery without clinical relevant differences. Similarly, responder rates for positive outcome were comparable between trainees with 92.9% and senior surgeons with 95.2% (p = 0.17). MIS THA seems to be a safe procedure during the learning curve of young orthopaedic specialists, but requires higher operative time.

## Introduction

Total hip arthroplasty (THA) is one of the most successful and frequently performed procedures in orthopaedic surgery^1^. For primary THA an increase of 174% is estimated in the United States by 2030^2^. The additional demand for arthroplasty surgeons is faced with a high number of expected retirements leading to a further shortfall of orthopaedic surgeons^3^. This development underlines the especial need of training young surgeons for performing THAs.

At the same time, although surgeons have always aimed for best operative results, the initiation of national joint registries, quality networks and online platforms urges hospitals to avoid patient dissatisfaction and complications in their statistical reports^4, 5^. Furthermore, the growing socioeconomic pressure for time efficient surgery additionally interferes with the opportunity for young surgeons to train operative procedures. However, the debate on outcome and complication rates after THA performed by trainees is still controversial^6–9^.

Over the last years, minimally invasive (MIS) surgical techniques with reduced incision lengths and less-extensive exposures have become more popular in THA. For experienced surgeons these approaches seem to be safe without greater operative complication rates in general^10^. However, for a trainee it might be more challenging to estimate the anatomic situation intraoperatively during MIS THA^11^. In literature, a prolonged operative time of eight minutes during the learning curve of an already in THA experienced surgeon compared to conventional THA has been described^12^. This study compared the first 86 consecutive MIS THAs with a matched cohort of hips treated with a standard lateral approach operated by one senior surgeon. At one year follow-up, both groups showed similar clinical outcome and complication rates. However, the effect of training young surgeons in MIS THA is still unknown.

In the current single centre study of over 1000 MIS THAs, we aimed to compare operative time, complication rates and early postoperative outcome within the first year after THA between trainees and senior surgeons using a MIS anterolateral hip approach at a university medical centre.

## Patients and Methods

A retrospective analysis of the institutional joint registry was performed. The local Ethics Commission waived approval. A power calculation was performed for investigation of the primary endpoint operative time. The corresponding hypothesis was tested on a 5% significance level. From a previous study analysing the learning curve of senior surgeons in MIS THA we set the effect size conservatively to 0.3^12^ with a sample ratio of 2:1. Based on these considerations, a sample size of 176 in the trainee group and 352 in the senior surgeon group achieved a power of 90% using two-sample t-tests (nQuery Advisor 7.0, Statistical Solutions Ltd, Cork, Ireland). From the database all patients undergoing primary cementless THA between June 2011 and December 2015 due to primary or secondary coxarthritis with complete pre- and postoperative outcome measures were chosen. During this period 2645 hip replacements were performed at the study centre including 327 revision arthroplasties of the hip. 1252 primary hip replacements met the inclusion criteria (primary THA, whole procedure performed by either senior surgeon or trainee, complete data files, complete pre and postoperative outcome measures). In 244 cases information on the operating team was incomplete. Patients undergoing revision THA or with incomplete data files or per-/postoperative outcome measures were excluded. A total of 1008 patients were included in final analysis. All operations were performed at our Department of Orthopedic Surgery at Regensburg University Medical Centre, Germany. Available data from the institutional joint registry included patient age, gender, date of admission and discharge, operative time, name of operating team, complication rates one year after surgery and pre- and one year postoperative Western Ontario and McMaster Universities Arthritis Index (WOMAC)^13^ and Euro-Qol 5D-5L (EQ-5D)^14^. The WOMAC is an international widely used score to evaluate outcome after total joint replacement representing a multidimensional measure of pain, stiffness and physical functional disability^15^. This measurement of outcomes by health-related quality of life questionnaire has especially been developed for patients with osteoarthritis and has been approved in several longitudinal studies with patients undergoing THA^16–18^. The EQ-5D is a widely used and tested descriptive instrument for evaluating health status. It defines health based on five dimensions: Mobility, Self-Care, Usual Activities, Pain/Discomfort and Anxiety/Depression. To improve the instrument’s sensitivity to small and medium health changes and to reduce ceiling effects the number of levels of severity in each dimension was expanded in 2005 to a five-level descriptive system increasing reliability and sensitivity of EQ-5D^14^.

**Figure 1:**
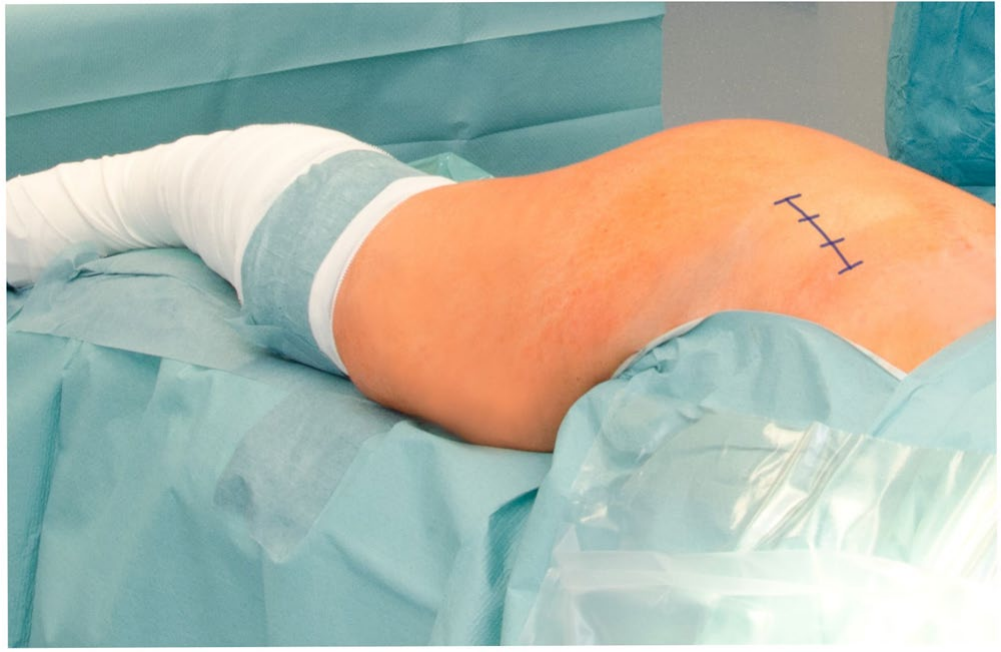
Patient position and skin incision of minimally invasive THA through an anterolateral approach in the lateral decubitus position.

All THAs were classified into two groups: Trainee THA and Senior THA. THA was defined as training procedure if the junior surgeon had completed the entire THA. All trainee operations were performed under the supervision of a senior surgeon according to the national guidelines. All trainees had a basic surgical education of 2 years prior to performing THAs. Therefore, trainees were between their 3rd to 5th year of surgical training. During the observed period of time 13 trainees performed the operations during their arthroplasty rotations. Experience in THA differed in the trainee group between 5 to 20 entirely performed MIS THAs. In contrast, each of the six senior surgeons had experience with more than 200 MIS THAs. Altogether 240 cases were available for trainee and 768 for senior THA, respectively. Anthropometric characteristics of the study group are shown in Table 1. Cementless THA was performed in the lateral decubitus position of all patients. A MIS single-incision anterolateral approach to the hip was used according to an intermuscular and interneural tissue plane between the tensor muscle and the gluteus medius muscle (Fig. 1)^19^. Press-fit acetabular components and cement-free hydroxyapatite-coated stems of one single manufacturer (Pinnacle^®^cup, Corail^®^stem or Trilock^®^stem, DePuy,Warsaw, IN, USA) with a femoral head size of 32 mm were used in all THAs.

**Table 1:** Anthropometric characteristics of the study group*.

N = 1008	Trainee	Senior
Number of THAs	N = 240	N = 768
Age (years)	65.9 ± 10.1	63.8 ± 10.8
Gender (men/women)	121/119	365/403
ASA-Class 1	27 (11.3%)	142 (18.5%)
ASA-Class 2	125 (52.1%)	411 (53.5%)
ASA-Class 3	85 (35.4%)	212 (27.6%)
ASA-Class 4	3 (1.3%)	3 (0.4%)
Operative time (min)	78.1 ± 25.4	69.3 ± 23.8
Length of hospital stay (d)	9.5 ± 1.8	9.4 ± 1.6

*For categorical data values are given as relative and absolute frequencies, for quantitative data values are given as mean (standard deviation), THA = total hip arthroplasty, ASA = American Society of Anaesthesiologists.

For dichotomizing responders and nonresponders within the first year after THA, the Outcome Measures in Rheumatology and Osteoarthritis Research Society International consensus responder criteria (OMERACT-OARSI) were used^13, 20^. These criteria assess responder status based on relative change in Index (WOMAC) scores in relation to benchmarks determined by expert consensus and statistical analyses. OMERACT-OARSI criteria were chosen since they do not depend on patient characteristics of the cohort and thus reducing any potential selection bias due to the retrospective design of the study^21^. The OMERACT-OARSI criteria to assess responders after THA include improvement in pain or function of at least 50% and absolute change of at least 20 points. Alternatively, responders are also defined by fulfilment of two of the following criteria: Improvement in pain of at least 20% and absolute change of at least 10 points, improvement in function of at least 20% and absolute change of at least 10 points, or global improvement of at least 20% with absolute change of at least 10 points^20^.

For statistical analysis, continuous data are presented as mean (standard deviation). Group comparisons were performed by two-sided t-tests. Absolute and relative frequencies were given for categorical data and compared between groups by chi-square tests. The primary hypothesis in the study was tested on 5% significance level. For all secondary hypotheses, significance levels were adjusted according to Bonferroni^22^. Multivariate logistic regression including age, gender, length of hospital stay, surgical experience, operative time, preoperative amount of pain medication and preoperative EQ-5D was performed to search for risk factors associated with responder rate within the first 12 months after THA. IBM SPSS Statistics 22 (SPSS Inc, Chicago, IL, USA) was used for analysis.

## Results

**Figure 2:**
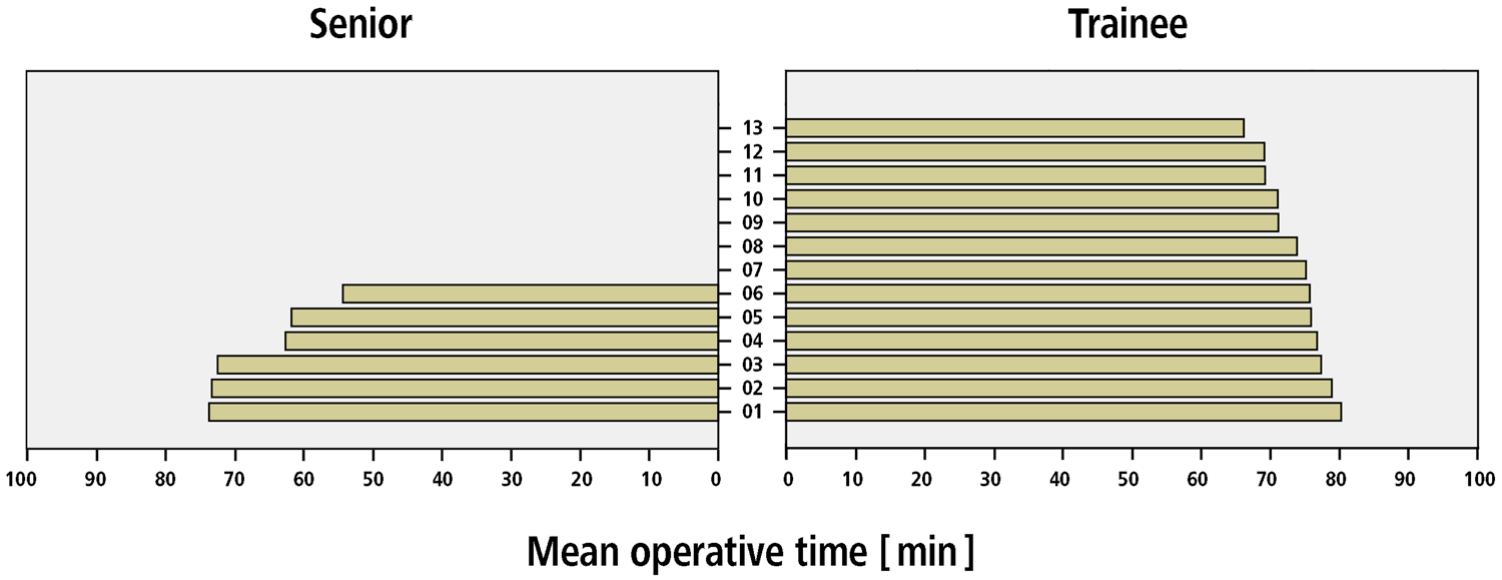
Number of THAs performed by each surgeon (six senior surgeons and 13 trainees) during the period of the study.

Mean operative time for MIS cementless THA was nine minutes longer for trainees compared to senior surgeons (78.1 ± 25.4 min versus 69.3 ± 23.8 min, p < 0.001, Fig. 2).

Complication rates in terms of intraoperative femur fractures (p = 0.84), postoperative dislocation (p = 0.21) and infection rates (p = 0.58) were comparable between the trainee and senior THA group (Table 2).

**Table 2:** Complication rates between the trainee and senior THAs*.

	Trainee	Senior	p-value
Intraoperative femur fractures	0.4% (1/240)	0.5% (4/768)	0.84
Dislocation	0.0% (0/240)	0.7% (5/768)	0.21
Joint infection	0.8% (2/240)	0.5% (4/768)	0.58

*For categorical data values are given as relative and absolute frequencies.

**Figure 3:**
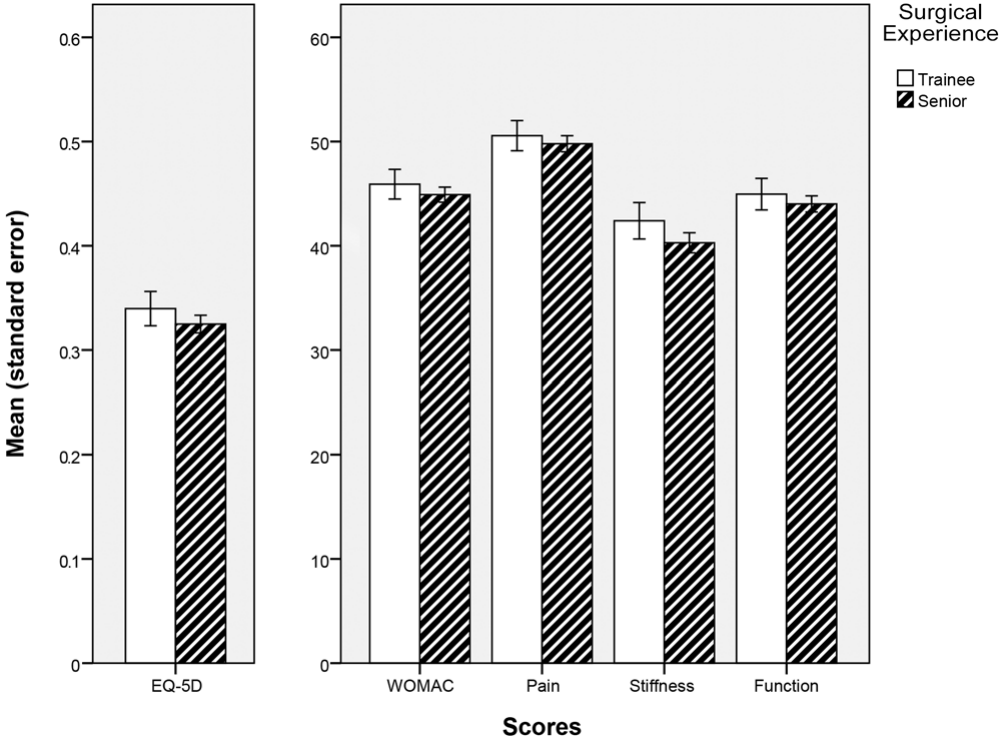
Improvement of patient reported outcome measures (WOMAC, EQ-5D) within the first year after THA in relation to surgical experience.

Patient reported outcome measures as assessed by WOMAC and EQ-5D showed excellent improvement within the first year postoperatively independently of trainee or senior surgeon performance (Fig. 3). At one year follow-up, we found no difference regarding WOMAC for MIS THAs operated by trainees (83.60 ± 17.18) or seniors (85.25 ± 15.84, p = 0.17). Correspondingly, EQ-5D values were similar between trainees (0.88 ± 0.17) and senior surgeons (0.89 ± 0.15, p = 0.17) one year postoperatively. Analysing outcome measures subscores, again one year results were comparable between trainee and senior THAs (Table 3).

**Table 3:** Western Ontario and McMaster Universities Arthritis Index (WOMAC) and Euro-Qol 5D-5L (EQ-5D) by surgeon grade preoperative and 1 year after minimally invasive THA*.

		EQ-5D preop	EQ-5D postop	WOMAC preop	WOMAC postop	Pain preop	Pain postop	Stiffness preop	Stiffness postop	Function preop	Function postop
Trainee	mean	0.54	0.88	37.70	83.60	37.02	87.58	38.33	80.73	37.82	82.77
	SD	0.22	0.17	18.16	17.18	20.24	16.41	22.88	20.51	19.06	17.91
Senior	mean	0.57	0.89	40.34	85.25	39.42	89.21	40.97	81.25	40.54	84.55
	SD	0.22	0.15	16.83	15.84	19.28	15.19	22.45	19.92	18.07	16.71
p-value		0.06	0.17	0.04	0.17	0.10	0.16	0.12	0.73	0.05	0.16

*For quantitative data values are given as mean (SD = standard deviation), preop = preoperative, postop = postoperative.

The rate of responders as defined by the OMERACT-OARSI criteria^20^ within the first year after THA was similar between the trainee group with 92.9% (223/240) and the senior surgeon group 95.2% (731/768, p = 0.17). Logistic regression analysis revealed and association between responder rate within the first year after MIS THA and preoperative high EQ-5D values (HR 0.09, 95%CI 0.02–0.45, p = 0.003), whereas surgical experience had no impact on outcome (Table 4).

**Table 4:** Multivariate analysis of risk factors associated with responder rate.

	HR	95% CI	P-value
Gender (male)	0.76	0.43–1.36	0.36
Age	0.99	0.96–1.01	0.31
ASA	1.13	0.74–1.75	0.57
Length of hospital stay	1.14	0.88–1.49	0.31
Surgical experience (Senior)	1.67	0.90–3.11	0.11
Operative time	1.00	0.99–1.01	0.97
Pain medication preop	1.00	0.99–1.00	0.22
EQ-5D preop	0.09	0.02–0.45	0.003

HR = Hazard Ratio, CI = Confidence Interval, preop = preoperative, ASA = American Society of Anaesthesiologists.

## Discussion

THA is one of the most performed but also increasingly costly operations within orthopaedic surgery worldwide^23^. In joint replacement, there is a learning curve and urgent need for educational and supervised operations for young surgeons^24–26^. Despite the increasing socioeconomic burdens in orthopaedic surgery, the training of young generations of surgeons remains crucial. Nowadays MIS techniques and surgical approaches have become more prevalent in THA^12^. We hypothesized that THAs performed by trainees would (1) consume more operative time (2) include higher complication rates and (3) produce worse outcome within the first year after MIS THA using an anterolateral approach compared to senior surgeons. Operative time for trainees was in general 9 minutes longer compared to senior surgeons. In contrast, all other hypotheses were not supported by our study.

There are several limitations of this study. First, the study design is a retrospective analysis. Due to the lack of randomization, trainees might have operated on easier cases compared to senior surgeons. Therefore, the results are susceptible to potential bias. To minimize potential selection bias we chose patient characteristics independent dichotomization for responders. Using non-cohort dependent benchmarks should maximize generalizability. Second, the current study is restricted to the information provided by the institutional joint registry. Other parameters such as the patient’s psychological or social status might have an impact on the patient specific outcome as well. Third, for the current analysis only short-term outcome data for the first 12 months were available. It would have been of interest to include long-term outcome and failure rates. A strength of the study is the fact that all data refer to one single University Medical Centre reflecting one specific operative as well as postoperative workflow. Similarly, components of a single THA manufacturer were used. All this contributes to minimizing confounding factors. Therefore, any results with regard to surgical experience is not due to surgical approach, intra- or postoperative treatment or the prosthetic component.

In answer to the first question of the study, the mean operative time for MIS THA was 9 minutes longer for trainees compared to senior surgeons. This is in accordance with literature reporting a prolonged operative time due to surgical training^9, 24, 27, 28^. In abdominal surgery resident involvement in laparoscopic appendectomy was related with an extension in surgical time of 14 minutes^27^. A previous study about training in THA showed a difference of 19 minutes between THAs performed by trainees (104 min) and trainers (85 min). This study included cemented and uncemented acetabular components using either a posterior or anterolateral approach^28^. In a different study an increase of 16 minutes (108 min versus 92 min) was observed for THAs by residents. The data were obtained from a database including information from different hospitals using different prosthetic designs and approaches^9^. Another study revealed a prolonged operative time of 11.5 (72.5 min versus 61.0 min) minutes for THA under teaching service compared to private service^24^. The authors qualified their results since parts of the difference in operative time might have been due to the difference of surgical technique with a higher rate of screw fixation of the cup in the teaching assistance group. Furthermore, this study assessed the participation of trainees in the operative procedure in general. This makes it hard to quantify how much of the THA was performed by the trainee and how much by the senior surgeon. We eliminated this variable by focusing on THAs entirely performed by trainees under supervision of a senior surgeon. From an economic point of view, a prolonged operative time of 9 minutes means higher financial expense for the hospital. In our cohort, according to the calculation of the administration the 230 trainee THAs resulted in an additional estimated expense of 33.000 $. An increase in perioperative resource consumption for teaching in total joint replacement of up to 22% has been reported in literature^25^. Therefore, teaching hospitals will need to seek for financial compensation from health care systems in order to train the next generation of surgeons.

In terms of our secondary outcome parameters, it was observed that the postoperative complications rates within one year of THA were low when compared with the literature^9^. There were no considerable differences between the trainee and senior surgeon group when compared the postoperative complication rates after THA. This finding is perfectly in accordance with the literature which describes that the resident involvement does not increase complication rates within the first 30 days after THA^9^. In contrast to our own results, a previous study observed a twofold increased risk of dislocation for THA performed by trainee surgeons. In this analysis, however, a posterior approach was used by unexperienced surgeons^7^. A different study focusing on the training in THA showed that the overall dislocation rate between the teaching service and the private practice was comparable until last follow up. It was reported that postoperative dislocation occurred in 8% of hips (10/119) in the teaching service and 6% (7/111) in the private practice. In addition, it was described that the mean follow-up time for the teaching service and the private practice was 59 months and 39 months, respectively. The head diameter in this study was mainly 28 mm limiting the comparability to our analysis^24^. More recently, a study reported that the long-term dislocation rate (ten years after THA) was 3.0% and this was not associated with experience of the surgeons. However, two different approaches were used in this study - (a) anterolateral, and (b) posterior. In addition, the study reported that a total of 1.5% of the patients developed deep wound infections within the period of ten years after THA^8^. In our study, the overall infection rate after one year of THA was 0.6%.

With the numbers available patient reported outcome measures as assessed by WOMAC and EQ-5D did not differ between the trainee and senior surgeon group. Both groups showed excellent improvement within the first year. The results are similar to previous published one-year results after THA^8, 24^. Accordingly, responders as defined by the OMERACT-OARSI criteria^20^ were not significantly different in relation to the surgical experience with a responder rate of over 90% in both groups. A multivariate analysis confirmed no association between responder rate and surgical intervention by trainees. Solely, high preoperative patient reported outcome measures correlated with nonresponders one year after THA. Similarly, other parameters such as operative time and length of hospital stay showed no correlation with the rate of responders after MIS THA. The results of the current analysis are in line with previous studies reporting patients with higher preoperative pain and better preoperative function are at high risk of worse outcome after joint replacement^29–31^. The association of better preoperative range of motion and worse postoperative function was confirmed in gait analysis. Nonresponders one year after THA showed a 26% higher range of motion preoperatively than responders^21^. In contrast to previous published studies^30, 32, 33^, patient age was not predictive for outcome in our analysis. Other parameters reported in literature to be associated with THA nonresponse such as educational level^33, 34^, comorbidities^30, 31, 35^ and preoperative expectations^29, 36^ were not available for the current study.

In conclusion, additional time is required to train the new orthopaedic generation in MIS THA. Therefore, training in MIS THA might be of economic relevance. The results from our study help to reduce the concerns that training in MIS THA might be automatically associated with worse outcome or higher complication rates within the first year postoperatively. Therefore, MIS THA under supervision seems to be a safe procedure during the learning curve of young surgeons

## References

[1] Learmonth, I. D., Young, C. & Rorabeck, C. The operation of the century: total hip replacement. *Lancet* **370**, 1508–1519, doi:10.1016/S0140-6736(07)60457-7 (2007). 10.1016/S0140-6736(07)60457-7 17964352

[2] Kurtz S, Ong K, Lau E, Mowat F, Halpern M (2007). Projections of primary and revision hip and knee arthroplasty in the United States from 2005 to 2030. J Bone Joint Surg Am.

[3] Fehring TK (2010). Joint replacement access in 2016: a supply side crisis. J Arthroplasty.

[4] Grimberg, A., Jansson, V., Liebs, T., Melsheimer, O. & Steinbrück, A. Endoprothesenregister Deutschland: Jahresbericht 2015. (2016).

[5] Weber P (2017). Does the certification according to EndoCert lead to a better quality of treatment?. Orthopade.

[6] Schoenfeld AJ, Serrano JA, Waterman BR, Bader JO, Belmont PJ (2013). The impact of resident involvement on post-operative morbidity and mortality following orthopaedic procedures: a study of 43,343 cases. Arch Orthop Trauma Surg.

[7] Hedlundh U, Ahnfelt L, Hybbinette CH, Weckstrom J, Fredin H (1996). Surgical experience related to dislocations after total hip arthroplasty. J Bone Joint Surg Br.

[8] Reidy MJ, Faulkner A, Shitole B, Clift B (2016). Do trainee surgeons have an adverse effect on the outcome after total hip arthroplasty?: a ten-year review. The bone & joint journal.

[9] Haughom BD, Schairer WW, Hellman MD, Yi PH, Levine BR (2014). Resident involvement does not influence complication after total hip arthroplasty: an analysis of 13,109 cases. J Arthroplasty.

[10] Reininga, I. H. *et al*. Minimally invasive and computer-navigated total hip arthroplasty: a qualitative and systematic review of the literature. *BMC Musculoskelet Disord* **11**, 92, doi:10.1186/1471-2474-11-92 (2010). 10.1186/1471-2474-11-92 PMC2879237 20470443

[11] Cheng T, Feng JG, Liu T, Zhang XL (2009). Minimally invasive total hip arthroplasty: a systematic review. Int Orthop.

[12] Sendtner E (2011). Tackling the learning curve: comparison between the anterior, minimally invasive (Micro-hip(R)) and the lateral, transgluteal (Bauer) approach for primary total hip replacement. Arch Orthop Trauma Surg.

[13] Bellamy N (1989). Pain assessment in osteoarthritis: experience with the WOMAC osteoarthritis index. Semin Arthritis Rheum.

[14] Herdman M (2011). Development and preliminary testing of the new five-level version of EQ-5D (EQ-5D-5L). Qual Life Res.

[15] Angst F, Aeschlimann A, Steiner W, Stucki G (2001). Responsiveness of the WOMAC osteoarthritis index as compared with the SF-36 in patients with osteoarthritis of the legs undergoing a comprehensive rehabilitation intervention. Annals of the rheumatic diseases.

[16] Quintana JM (2005). Responsiveness and clinically important differences for the WOMAC and SF-36 after hip joint replacement. Osteoarthritis Cartilage.

[17] March L (2002). Cost of joint replacement surgery for osteoarthritis: the patients’ perspective. J Rheumatol.

[18] Jones CA, Voaklander DC, Johnston DW, Suarez-Almazor ME (2001). The effect of age on pain, function, and quality of life after total hip and knee arthroplasty. Arch Intern Med.

[19] Michel MC, Witschger P (2007). MicroHip: a minimally invasive procedure for total hip replacement surgery using a modified Smith-Peterson approach. Ortop Traumatol Rehabil.

[20] Pham T (2004). OMERACT-OARSI initiative: Osteoarthritis Research Society International set of responder criteria for osteoarthritis clinical trials revisited. Osteoarthritis Cartilage.

[21] Foucher KC (2017). Preoperative gait mechanics predict clinical response to total hip arthroplasty. J Orthop Res.

[22] Abdi, H. Bonferroni and Sidak corrections for multiple comparisons. (2007).

[23] Bozic KJ, Beringer D (2007). Economic considerations in minimally invasive total joint arthroplasty. Clin Orthop Relat Res.

[24] Woolson ST, Kang MN (2007). A comparison of the results of total hip and knee arthroplasty performed on a teaching service or a private practice service. J Bone Joint Surg Am.

[25] Lavernia, C. J., Sierra, R. J. & Hernandez, R. A. The cost of teaching total knee arthroplasty surgery to orthopaedic surgery residents. *Clin Orthop Relat Res*, 99–107 (2000). 10.1097/00003086-200011000-00014 11064979

[26] Weber M (2017). Total knee arthroplasty in the elderly. Orthopade.

[27] Advani, V., Ahad, S., Gonczy, C., Markwell, S. & Hassan, I. Does resident involvement effect surgical times and complication rates during laparoscopic appendectomy for uncomplicated appendicitis? An analysis of 16,849 cases from the ACS-NSQIP. *Am J Surg* **203**, 347–351; discussion 351–342, doi:10.1016/j.amjsurg.2011.08.015 (2012). 10.1016/j.amjsurg.2011.08.015 22364902

[28] Palan J (2009). The trainer, the trainee and the surgeons’ assistant: clinical outcomes following total hip replacement. J Bone Joint Surg Br.

[29] Pinto PR, McIntyre T, Ferrero R, Almeida A, Araujo-Soares V (2013). Risk factors for moderate and severe persistent pain in patients undergoing total knee and hip arthroplasty: a prospective predictive study. PLoS One.

[30] Nilsdotter AK, Petersson IF, Roos EM, Lohmander LS (2003). Predictors of patient relevant outcome after total hip replacement for osteoarthritis: a prospective study. Annals of the rheumatic diseases.

[31] Hawker GA (2013). Which patients are most likely to benefit from total joint arthroplasty?. Arthritis Rheum.

[32] Nilsdotter AK, Lohmander LS (2002). Age and waiting time as predictors of outcome after total hip replacement for osteoarthritis. Rheumatology (Oxford).

[33] Judge A, Cooper C, Williams S, Dreinhoefer K, Dieppe P (2010). Patient-reported outcomes one year after primary hip replacement in a European Collaborative Cohort. Arthritis care & research.

[34] Greene ME (2014). Education attainment is associated with patient-reported outcomes: findings from the Swedish Hip Arthroplasty Register. Clin Orthop Relat Res.

[35] Judge A (2012). Clinical tool to identify patients who are most likely to achieve long-term improvement in physical function after total hip arthroplasty. Arthritis care & research.

[36] Judge A (2011). Assessing patients for joint replacement: can pre-operative Oxford hip and knee scores be used to predict patient satisfaction following joint replacement surgery and to guide patient selection?. J Bone Joint Surg Br.

